# Application of Potential Phosphate-Solubilizing Bacteria and Organic Acids on Phosphate Solubilization from Phosphate Rock in Aerobic Rice

**DOI:** 10.1155/2013/272409

**Published:** 2013-10-03

**Authors:** Qurban Ali Panhwar, Shamshuddin Jusop, Umme Aminun Naher, Radziah Othman, Mohd Ismail Razi

**Affiliations:** ^1^Department of Land Management, Faculty of Agriculture, Universiti Putra Malaysia, 43400 Serdang, Selangor, Malaysia; ^2^Institute of Tropical Agriculture, Universiti Putra Malaysia, 43400 Serdang, Selangor, Malaysia; ^3^Bangladesh Rice Research Institute, Gazipur, Bangladesh

## Abstract

A study was conducted at Universiti Putra Malaysia to determine the effect of phosphate-solubilizing bacteria (PSB) and organic acids (oxalic & malic) on phosphate (P) solubilization from phosphate rock (PR) and growth of aerobic rice. Four rates of each organic acid (0, 10, 20, and 30 mM), and PSB strain (*Bacillus* sp.) were applied to aerobic rice. Total bacterial populations, amount of P solubilization, P uptake, soil pH, and root morphology were determined. The results of the study showed significantly high P solubilization in PSB with organic acid treatments. Among the two organic acids, oxalic acid was found more effective compared to malic acid. Application of oxalic acid at 20 mM along with PSB16 significantly increased soluble soil P (28.39 mg kg^−1^), plant P uptake (0.78 P pot^−1^), and plant biomass (33.26 mg). Addition of organic acids with PSB and PR had no influence on soil pH during the planting period. A higher bacterial population was found in rhizosphere (8.78 log_10_ cfu g^−1^) compared to the nonrhizosphere and endosphere regions. The application of organic acids along with PSB enhanced soluble P in the soil solution, improved root growth, and increased plant biomass of aerobic rice seedlings without affecting soil pH.

## 1. Introduction

Phosphate-solubilizing bacteria play a vital role in P solubilization by producing organic acids. Additionally, soils contain low molecular weight organic acids with one or more carboxylic groups and some of these acids like citrate, oxalate, acetate, malate, isocitrate, and tartrate. Plant root exudates and microorganisms produce organic acids and degrade complex organic molecules [1, 2]. Organic acids perform many functions in the soil, such as root nutrient acquisition, mineral weathering, microbial chemotaxis, and metal detoxification. They play an important role in the mobilization of soil P and enhance P bioavailability [3, 4] with decreasing P adsorption and dissolution of insoluble P compounds such as Ca, Fe, and Al phosphates [3]. Organic acid exudation from roots is considered an important mechanism for plants to adapt in P-deficient environments [5]. The mechanism involves mobilization of unavailable P in the soil by organic acids [6]. The role of organic acids in P solubilization is highly soil dependent. On the contrary, one of the P solubilization mechanisms of microbes is the production of organic acids [7]. A number of organic acids such as lactic, citric, 2-ketogluconic, malic, oxalic, malonic, tartaric, and succinic have been identified that have chelating properties [8]. Evidence showed that addition of organic acids to soils increased plant P uptake [9]. 

 The PR is an alternative and natural source of P. The dissolution of P from PR is required for the availability of P. Many factors affect the dissolution of PR in soil such as, chemical composition, particle size of the PR, soil characteristics, pH, H_2_PO_4_^−^, and Ca_2_^+^ [10]. Dissolution of PR in acid soils can be explained as follows Khasawneh and Doll [11]:

(1) $$\begin{array}{c} {\text{Ca}}_{10}{\left({\text{PO}}_{4}\right)}_{6}{\text{F}}_{2}+12{\text{H}}^{+}\Rightarrow 10{{\text{Ca}}_{2}}^{+}+6{\text{H}}_{2}{\text{P}{\text{O}}_{4}}^{-}+2{\text{F}}^{-}. \end{array}$$

The equation specifies that the rate of dissolution is determined by the concentration of protons (H^+^) and the concentration of reaction products of Ca_2_^+^ and H_2_PO_4_. In acidic soils, improvement of P nutrition to plants by direct application of PR as P fertilizer has been considered [12]. Phosphate rocks are affluent in calcium phosphate complexes and are soluble in acidic soil environments. Either microbial released organic acids or any acidic condition may favor P solubilization from PR. Hence, the present study was undertaken to determine the effect of different rates of organic acids with phosphate-solubilizing bacteria on P solubilization and their effect on growth of aerobic rice.

## 2. Materials and Methods

The experiment was conducted under *in vitro *condition. PSB16 (*Bacillus* sp.) isolated form aerobic rice rhizosphere [13], which was tested to produce indoleacetic acid, P solubilization, organic acids, and siderophore production in *in vitro *condition. The isolated strain was identified using 16S rRNA gene sequencing with accession number JX103827. Four rates each of oxalic and malic acids (0, 10, 20, and 30 mM) and PR (Christmas Island Rock Phosphate) at 60 kg P_2_O_5_ ha^−1^ were applied. The aerobic rice (*var* M9) was grown in the growth chamber for 40 days. Total bacterial populations, P solubilization, soil pH, root morphology, and agronomic parameters were recorded after 40 days of growth. 

### 2.1. Seed Surface Sterilization, Inoculation, and Growth of Rice Seedlings

The surface sterilized seven days old seedlings were transplanted into pots containing sterilized soil (500 g) with 4 uniform seedlings per pot. Plants were grown for 40 days in a growth chamber with 12 h light/dark cycle at 29 ± 1°C temperature. Approximately 5 × 10^9^ mL^−1^ of live washed bacterial cells of *Bacillus* sp. (PSB16) were used as inoculum in each bacterial treatment. 

### 2.2. Determination of Bacterial Population, Plant Biomass, and Plant Tissue P

The total bacterial population was determined from rhizosphere, nonrhizosphere, and endosphere of aerobic rice plants. After harvest, soil available P was determined using Bray 2 [14] and total plant tissue P was analyzed by the wet digestion method [15].

### 2.3. Determination of Root Development

The root length (cm), total surface (cm^2^), and root volume (cm^3^) were quantified using a scanner (Expression 1680, Epson) equipped with a 2 cm depth plexiglass tank (20 × 30 cm) filled with UP H_2_O [16]. 

### 2.4. Data Analysis

The experiment was conducted with the three factors in four replicates in a completely randomized design. Data obtained were statistically analyzed using the SAS software program (Version 9.2), and treatment means were compared using Tukey's test (*P* < 0.05).

## 3. Results 

### 3.1. Effect of Organic Acids and PSB on P Solubilization and Plant P Uptake

**Figure 1:**
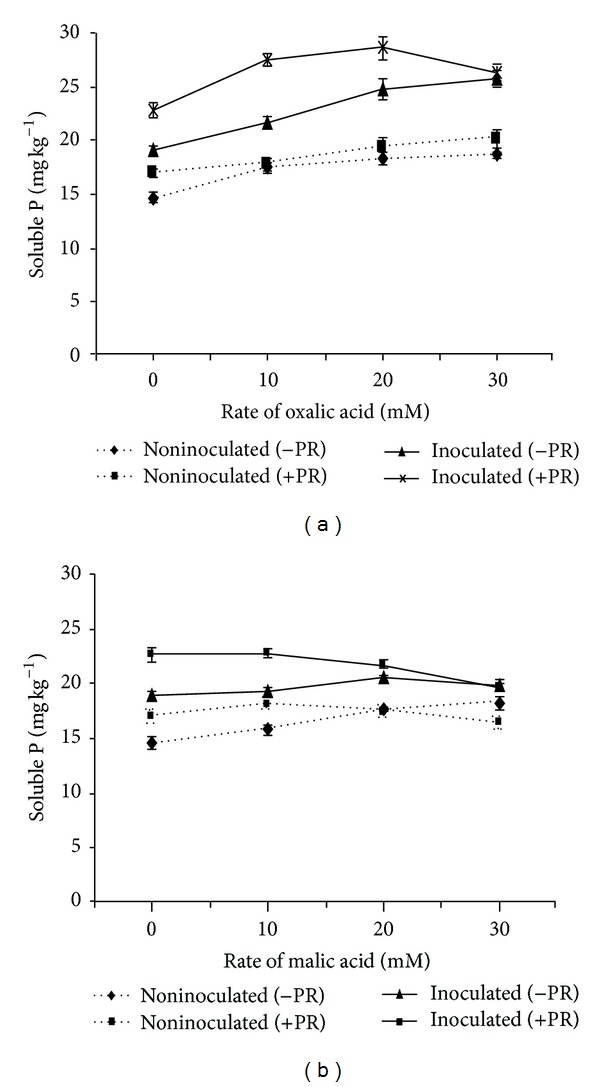
Effect of (a) oxalic acid (b) malic acid on P solubilization. Bars indicate standard error, *n* = 5.

Higher values of solubilized P were found in PSB inoculated treatments with organic acids application. Significantly high amount of solubilized P (31.51%) was found in PSB inoculated treatments with 20 mM oxalic acid (Figure 1(a)). 

 Between the two acids, P solubilization was found higher in oxalic compared to malic acid at various rates. In malic acid treatments, higher P solubilization (21.57 mg kg^−1^) was observed with 10 mM of malic acid in PSB inoculated treatments (Figure 1(b)). The amount of P solubilization and plant P uptake differed with the bacterial inoculation and rate of organic acid application. 

 The application of organic acids also affected plant P uptake. Significantly higher plant P uptake was observed in PSB inoculated treatments added with organic acids (Table 1). Among both, the highest values of plant P uptake was found in PSB with oxalic acid at 20 mM (0.78 P pot^−1^) followed by 30 mM (0.75 P pot^−1^) concentrations, respectively. 

### 3.2. Effect of Organic Acids on Bacterial Populations

**Figure 2:**
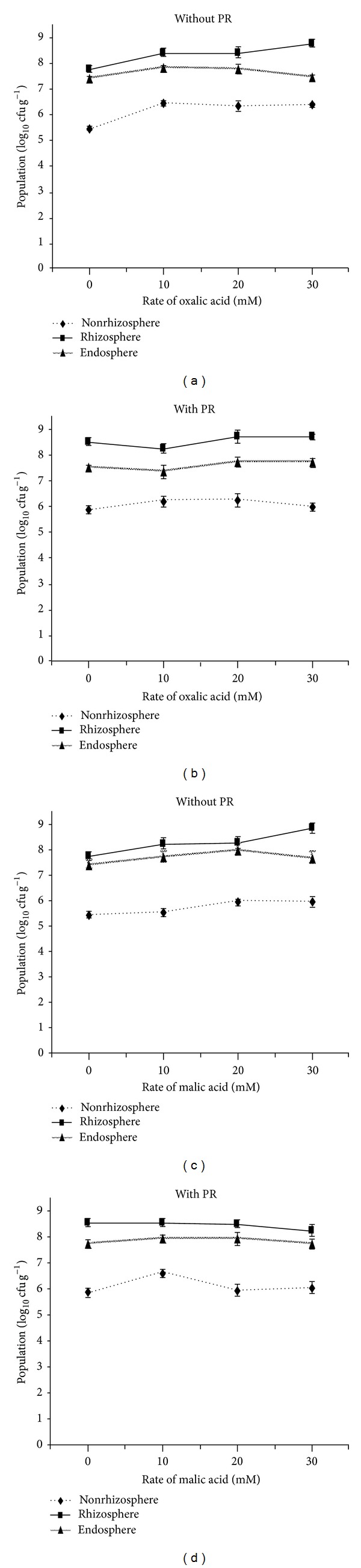
Effect of organic acids on PSB16 population, (a) oxalic acid without PR, (b) oxalic acid with PR, (c) malic acid without PR, (d) malic acid with PR. Bars indicate standard error, *n* = 5.

The addition of organic acids influenced the bacterial population. Significantly (*P* < 0.05) higher populations were found in the rhizosphere (8.78 log_10_ cfu g^−1^), while lower populations were found in nonrhizosphere soils (5.40 log_10_ cfu g^−1^). The highest rhizosphere population (8.65 log_10_ cfu g^−1^) was found in the 30 mM oxalic acid treatment (Figures 2(a) and 2(b)). In the case of malic acid, a significantly higher population was found in the rhizosphere at 30 mM acid without PR treatment (8.78 log_10_ cfu g^−1^), whereas, in nonrhizosphere soil the highest population was recorded at 10 mM acid without PR (6.54 log_10_ cfu g^−1^). However, the endosphere population was not influenced by the addition of organic acids (Figures 2(c) and 2(d)). Solubilization of P by PSB in the rhizosphere is a continuous process. The addition of organic acids had a positive influence on the PSB16 population. A higher population was found with the addition of organic acids combined with PR. The changes in PSB population occurred mostly in the nonrhizosphere region, and it becomes lower with the addition of oxalic acid treatments. 

### 3.3. Effect of Organic Acids and PSB on Plant Height and Plant Biomass

There were significant differences found between the organic acids and their various rates. Significantly (*P* < 0.05) high values of plant height were observed in the PSB inoculated treatments. Among both acids, the highest plant height (23 cm) was observed in oxalic acid (20 mM) with PR and PSB16, compared to malic acid (Table 2). 

**Figure 3:**
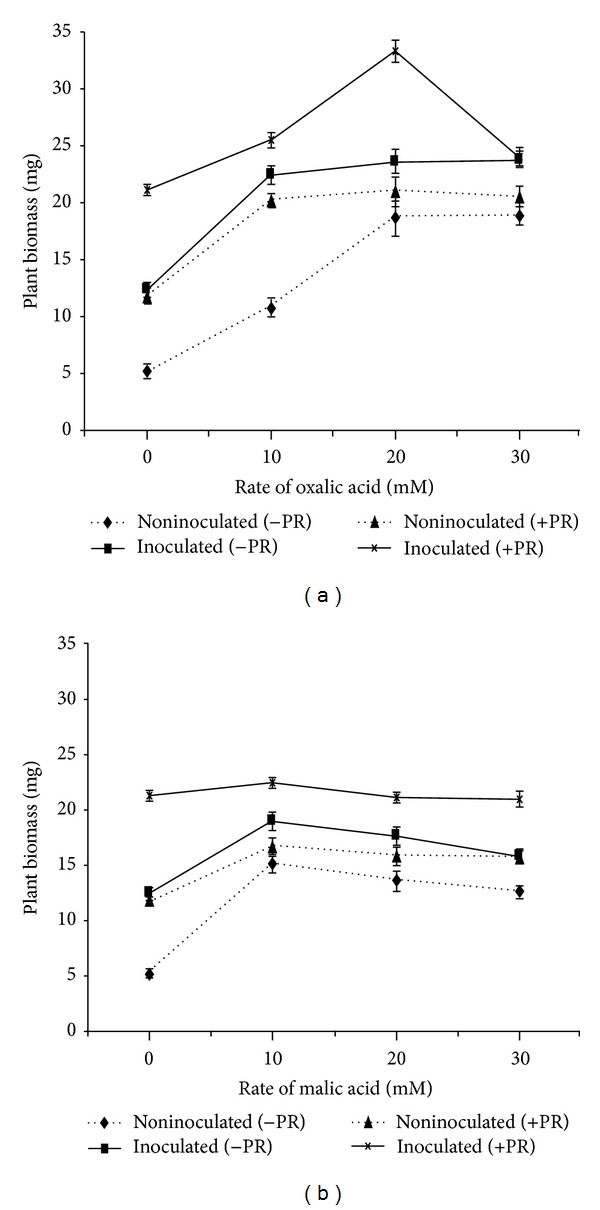
Effect of organic acids (a) oxalic acid (b) malic acid on plant biomass. Bars indicate standard error, *n* = 5.

 Inoculation of PSB with PR showed higher plant biomass than noninoculated treatments (Figure 3). Application of organic acids with PSB16 and PR significantly increased the plant biomass, and comparatively oxalic acid produced higher plant biomass than malic acid. The highest plant biomass (33.26 mg) was recorded in PSB inoculated plants at 20 mM oxalic acid concentration with PR (Figure 3(a)). Application of malic acid increased biomass at 10 mM and showed no further increase at higher levels (Figure 3(b)). 

### 3.4. Effect of Organic Acids and PSB on Soil pH

**Figure 4:**
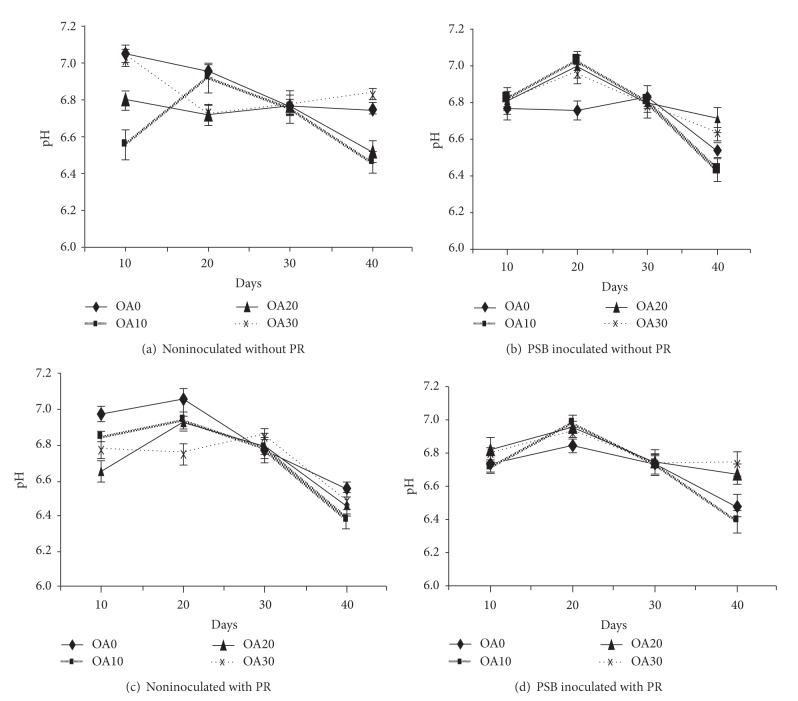
Effect of oxalic acid (OA) on soil pH. Bars indicate standard error, *n* = 5.

**Figure 5:**
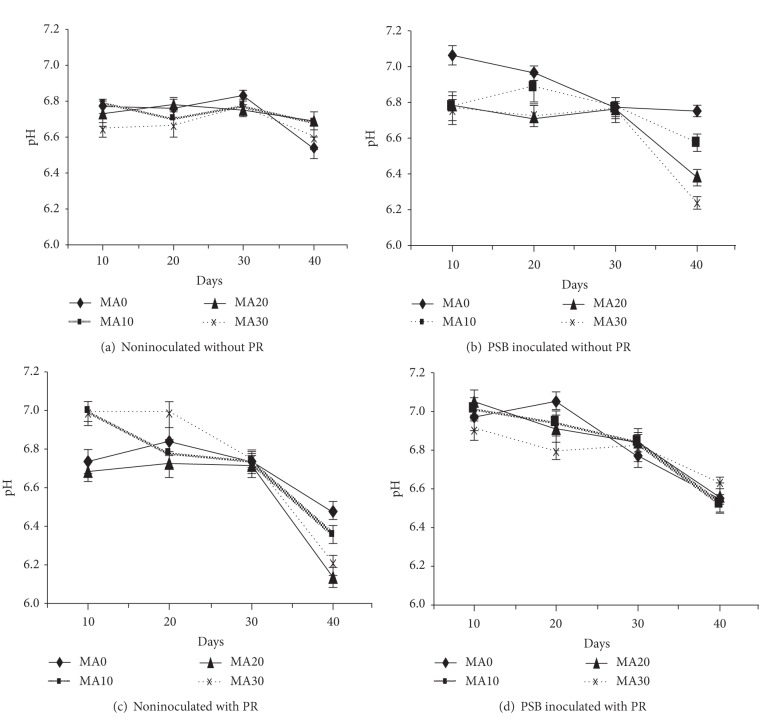
Effect of malic acid (MA) on soil pH. Bars indicate standard error, *n* = 5.

The soil pH during the planting period was not much affected (Figures 4 and 5). Slightly lower pH values were observed in PSB inoculated compared to noninoculated treatments. Among both acids a higher decrease in pH values was found with the addition of malic acid with PSB inoculation. The instability of organic acid or soil buffering system might have an effect in regulating the soil pH. However, slight decreases were found which could be due to the influence of organic acids to change pH in the rhizospheric regions. 

### 3.5. Effect of Organic Acids and PSB on Root Development

**Figure 6:**
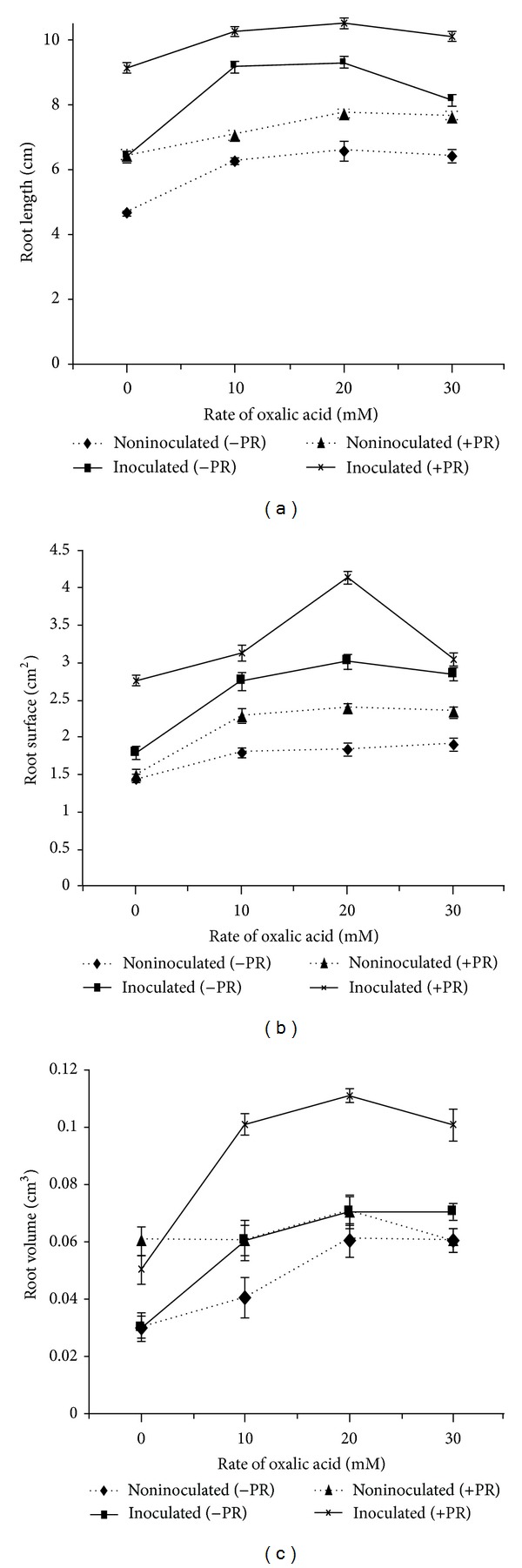
Effect of oxalic acid on (a) root length, (b) root surface, and (c) Root volume. Bars indicate standard error, *n* = 5.

**Figure 7:**
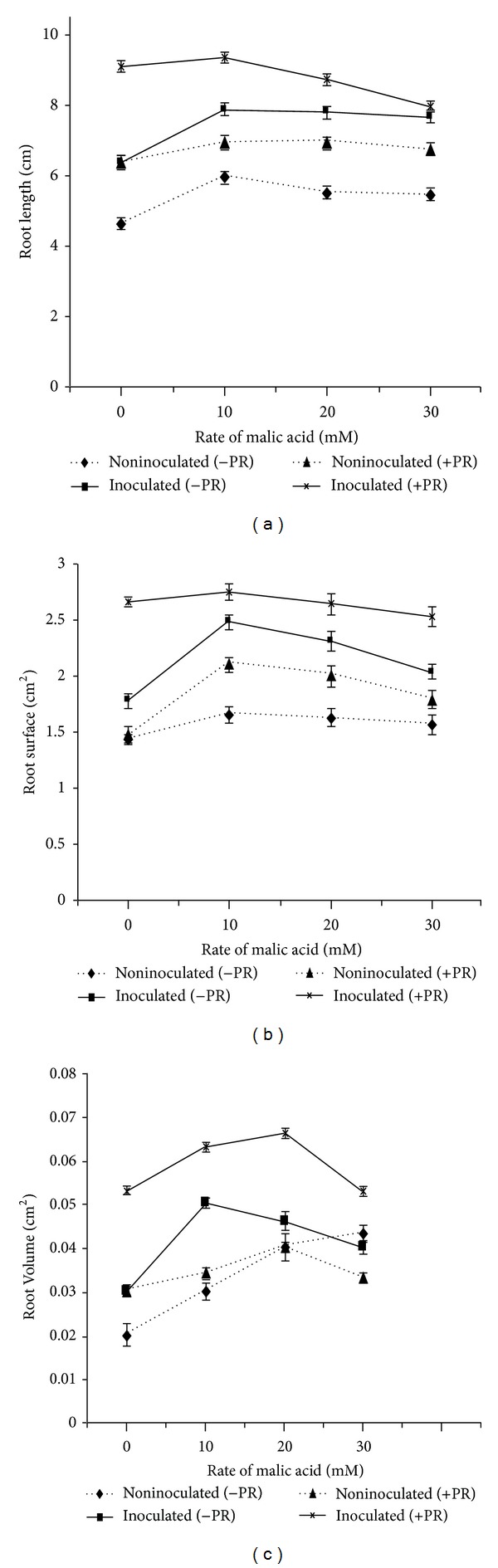
Effect of malic acid on (a) root length, (b) root surface, and (c) root volume. Bars indicate standard error, *n* = 5.

Root development in aerobic rice was influenced by the application of organic acids, PSB, and PR. The highest root length, surface area, and root volume were found in treatments with organic acids, PR, and PSB16 inoculations (Figures 6 and 7). Among both acids, oxalic acid produced higher root growth. Significantly (*P* < 0.05) higher root length (7.64 cm), root surface area (2.36 cm^2^) and root volume (0.11 cm^3^) were found in oxalic acid at 20 mM with PR and PSB16 inoculated treatments (Figures 6 and 7). External application of organic acids along with PSB enhanced soluble P in the solution and this had a positive impact on root growth. 

## 4. Discussion 

The PSB and organic acid solubilized higher values of P in aerobic rice. However, high amounts of solubilized P were observed in PSB inoculated with oxalic acid application. These results are in agreement with the findings of Asea et al. [17] who noted that an application of oxalic acid was effective for P solubilization. Similar results were observed by Wei et al. [18] who observed that oxalic acids are more prominent in solubilizing P compared to other organic acids. Strong binding abilities of oxalic and citric acids have been proven as the most competent agents to solubilize soil P [1]. The application of organic acids also affected plant P uptake.

The bacterial population was influenced with the addition of organic acids. The population was varied in both organic acids at different rates in plant rhizosphere and non-rizosphere population. Thus, it might be due direct contact of the acids in soil and could be able to perform prominently for the P solubilization. Moreover, solubilization of P by PSB in the rhizosphere is a continuous process. PSB solubilize insoluble P by several mechanisms such as acidification, chelation, and exchange reactions [19]. It was reported that PSB solubilized PR as well as di-calcium phosphate and about 20–50 times less organic acids secreted by PSB were required for P solubilization. Furthermore, PSB strains (*Citrobacter koseri *and *Bacillus coagulans*) have been proven to solubilize PR with many organic acids [20]. The release of P is extremely soil dependent with higher concentrations of organic acids required to mobilize major quantities of P into the soil solution [21]. 

The changes in PSB population occurred mostly in the nonrhizosphere region, and it becomes lower with the addition of oxalic acid treatments. This could happen due to the transfer of bacteria from the nonrhizosphere to the rhizosphere zone, as the rhizospheric zone is a source of organic carbon which is needed for microbial activity. Cannon et al. [22] pointed out that there was a significant increase in soil and plant tissue P where oxalic acid was applied and is readily degraded by microorganisms. The increase in soluble P in the solution and increase in plant biomass proved that application of organic acids along with PSB16 had a positive effect on plant and microbial growth.

The organic acids with PSB16 and PR increased the plant biomass. Besides P solubilization activity, PSB liberates phytohormone (IAA) that might have an influence on root growth. The extensive root system increased nutrient uptake from the surroundings which increased plant biomass [23]. The organic acids serve as a source of carbon for the microorganisms, and subsequently, affect the rhizosphere microbial population as well as plant growth [24]. 

The application of organic acid, PSB16, and PR slightly affected the soil pH values. This could be due to the soil buffering system, and it did not affect much the change of soil pH. The slight reductions were observed in the rhizosphere, that could be due to the influence of organic acid applications. These results are consistent with the findings of Zeng et al. [25] who reported that the organic acid have significant positive correlation pH of the rhizosphere of rice plants furthermore, when PR is added to soil (alfisols), mostly organic acids brought about a drop in pH for P released [26]. 

The plant root development in aerobic rice was affected by the application of organic acids, PSB, and PR. External application of organic acids along with PSB enhanced soluble P in the solution and this had a positive impact on root growth. These results are in agreement with the findings of Srivastava et al. [27] who reported that addition of organic acid with PR brought release of P and showed positive effect on plant growth. Moreover, Hoffland et al. [28] found organic acids in root exudates which were highly efficient in increasing P release from PR. The root development and plant biomass were correlated with the higher availability of P; moreover, PSB application may also have some other beneficial effects like phytohormones production.

## 5. Conclusion

The application of organic acids and PSB16 showed differences in P solubilization from PR. Among both acids, oxalic acid showed better results when compared to malic acid. The PSB16 population and soil pH were not affected by the application of organic acids. Application of oxalic acid at 20 mM along with PSB16 significantly increased soluble P release from PR. In conclusion, addition of organic acids with PSB increased the solubility of PR and had a significant effect on the growth of aerobic rice. However, at the higher concentration of oxalic acid application may present a health risk, especially for children. 

## Figures and Tables

**Table 1:** Effect of organic acids on plant P uptake.

Dose of organic acid (mM)	Plant P uptake (mg P pot^−1^)							
	Oxalic acid				Malic acid			
	PSB noninoculated Treatments		PSB inoculated Treatments		PSB noninoculated Treatments		PSB inoculated Treatments	
	Rate of PR (kg ha^−1^)							
	0	60	0	60	0	60	0	60
0	0.21e	0.29b	0.25f	0.36c	0.21d	0.28b	0.23e	0.33c
10	0.24d	0.30b	0.29e	0.48b	0.22d	0.29b	0.25d	0.39a
20	0.27c	0.37a	0.32d	0.78a	0.24cd	0.34a	0.27d	0.37ab
30	0.28bc	0.39a	0.37c	0.75a	0.26c	0.36a	0.32c	0.36b

Means within the same column followed by the same letters are not significantly different at *P* ≤ 0.05.

**Table 2:** Effect of organic acids on plant height of aerobic rice seedling.

Dose of organic acid (mM)	Plant height (cm)							
	Oxalic acid				Malic acid			
	PSB noninoculated Treatments		PSB inoculated Treatments		PSB noninoculated Treatments		PSB inoculated Treatments	
	Rate of PR (kg ha^−1^)							
	0	60	0	60	0	60	0	60
0	12.83c	17ab	16c	20b	12.83d	17a	16c	20a
10	15b	19a	21b	22ab	14.67c	18a	19a	20a
20	17ab	19a	21.3b	23a	16b	17a	17b	19.33a
30	17ab	18.33a	21b	21b	16.33ab	17a	17.67b	19.30a

Means within the same column followed by the same letters are not significantly different at *P* ≤ 0.05.
